# Identifying latent subgroups in the older population seeking primary health care for a new episode of back pain – findings from the BACE-N cohort

**DOI:** 10.1186/s12891-024-07163-0

**Published:** 2024-01-13

**Authors:** Lise Kretz Grøndahl, Iben Axén, Silje Stensrud, Trynke Hoekstra, Ørjan Nesse Vigdal, Rikke Munk Killingmo, Kjersti Storheim, Margreth Grotle

**Affiliations:** 1 Department of Rehabilitation Science and Health Technology, Oslo Metropolitan University, Oslo, Norway; 2 Et Liv i Bevegelse, the Norwegian Chiropractors’ Research Foundation, Oslo, Norway; 3 Unit of Intervention and Implementation for Worker Health, Institute of Environmental Medicine, Karolinska Institutet, Stockholm, Sweden; 4 Department of Health Sciences, Faculty of Science, Amsterdam Public Health Research Institute, Vrije Universiteit Amsterdam, Amsterdam, The Netherlands; 5 Department of Research and Innovation, Division of Clinical Neuroscience, Oslo, Norway

**Keywords:** Back pain, Older adult, Latent class

## Abstract

**Background:**

Back pain is the number one condition contributing to years lived with disability worldwide, and one of the most common reasons for seeking primary care. Research on this condition in the ageing population is sparse. Further, the heterogeneity of patients with back pain complicates the management in clinical care. It is possible that subgrouping people with similar characteristics would improve management. This paper aimed to identify latent classes based on demographics, pain characteristics, psychosocial behavior, and beliefs and attitudes about back pain, among older patients seeking primary care with a new episode of back pain, and to examine if there were differences regarding the classes’ first point-of-contact.

**Methods:**

The study was part of the international BACE (Back complaints in elders) consortium and included 435 patients aged ≥ 55 years seeking primary care (general practitioners, physiotherapists, and chiropractors) in Norway from April 2015 to March 2020. A latent class analysis was performed to identify latent classes. The classes were described in terms of baseline characteristics and first point-of-contact in primary care.

**Results:**

Four latent classes were identified. The mean age was similar across groups, as were high expectations towards improvement. Class 1 (*n* = 169, 39%), the “positive” class, had more positive attitudes and beliefs, less pain catastrophizing and shorter duration of current pain episode. Class 2 (*n* = 31, 7%), the “fearful” class, exhibited the most fear avoidance behavior, and had higher mean pain intensity. Class 3 (*n* = 33, 8%), the “distressed” class, had the highest scores on depression, disability, and catastrophizing. Finally, class 4 (*n* = 202, 46%), the “hopeful” class, showed the highest expectations for recovery, although having high pain intensity. The identified four classes showed high internal homogeneity, sufficient between-group heterogeneity and were considered clinically meaningful. The distribution of first point-of-contact was similar across classes, except for the positive class where significantly more patients visited chiropractors compared to general practitioners and physiotherapists.

**Conclusions:**

The identified classes may contribute to targeting clinical management of these patients. Longitudinal research on these latent classes is needed to explore whether the latent classes have prognostic value. Validation studies are needed to evaluate external validity.

**Trial registration:**

Clinicaltrials.gov NCT04261309.

**Supplementary Information:**

The online version contains supplementary material available at 10.1186/s12891-024-07163-0.

## Background

Musculoskeletal (MSK) conditions are highly prevalent and represent a considerable burden on the society as well as for the individual [1]. Among these conditions, low back pain (LBP) is the highest rated cause of years lived with disability globally [2], with 70–85% of the population estimated to experience an episode of low back pain (LBP) at some point in their lives [3]. However, most previous research has focused on younger people, often in their productive ages [4, 5]. The anatomical and physiological explanations and functional consequences of LBP in the older population are not comparable to those in the working population [6]. Some studies have reported that the geriatric population has a higher prevalence of severe, persistent and disabling pain [7, 8] compared to the younger adults, with low back pain being one of the most frequently reported symptoms causing functional limitations and disability [9]. Consequently, with a decline in physical activity and function, back pain may represent a major health burden for older individuals. Considering the ageing of the population globally, this expanding problem represents a considerable challenge for health care systems in the future.

Back pain is predominantly managed in primary care by first contact clinicians such as general practitioners (GP), physiotherapists (PT) and chiropractors (DC). Personal factors (age, sex, educational level and employment status) [10–13], pain characteristics [11, 14, 15], functional level [10], and psychological and behavioral characteristics (fear avoidance and expectations) [11] are factors found to be associated with which health care provider the patient seeks for their back pain. However, previous research has found demographic differences among patients seeking care depending on their first point of contact. Patients seeking chiropractic care are well educated, working, and report better health related quality of life, while individuals seeing their family physician have lower socioeconomic status [16]. This is, however, not investigated extensively among the elderly [17].

A plethora of treatment options are provided [18], but in terms of reducing pain and improving function, the effectiveness of these various treatment options remains moderate at best [18]. Management is complicated by heterogeneity among patients [19]. Identifying homogeneous patient groups could be useful in developing targeted interventions to improve treatment outcomes. Previous research have classified patients according to diagnosis [20–26], a single variable like e.g. pain-site [27, 28], or psychological dimensions [25]. Recently, researchers have called for studies subgrouping patients across multidimensional factors (i.e. psychological, behavioral, and social) in the population experiencing MSK pain [29].

Latent Class Analysis (LCA) has the potential to identify subgroups that are homogenous in their baseline clinical presentation based on similar patterns of responses to the questionnaire items [30]. Such subgrouping has shown promise in populations with MSK pain, and these subgroups might facilitate better prognostic estimates and more targeted treatment [29]. A study among patients with low back pain found LCA classes with prognostic capacity [31], but a recent Danish study found that LCA-derived subgroups provided little prognostic value [24]. Few studies have investigated if there are hidden patterns or underlying subgroups among MSK patients based on a broad set of prognostic factors across the biopsychosocial domains, and to the best of our knowledge, none has investigated the elderly.

This study aimed to identify homogenous subgroups among patients aged 55 or older seeking primary care for a new episode of back pain. The variables chosen for the analysis were based on previous prognostic research, and included pain characteristics [32–34], psychosocial factors [32, 35, 36], beliefs [33, 34] and attitudes about back pain [37], function [33], and comorbidities [34]. A second aim was to investigate if the identified classes differed in terms of type of health care provider (i.e. GP, PT, or DC) the patients first contacted for their back pain.

## Method

### Design and setting

This study used cross-sectional (baseline) data from the Back Complaints in the Older adult -Norway (BACE-N); a prospective observational cohort study of older adults seeking primary health care in primary care in Norway for a new episode of back pain [38]. The Norwegian Social Science Data Service approved this study (reference no. 42419) and this study did not need ethics approval as treatment was not affected by participation (Norwegian Regional Committee for Medical Research Ethics, ref. no 2014/1634/REK vest).

### Study sample and recruitment

Eligible patients for the BACE-N study were women and men, 55 years of age or older who sought primary care (GP, PT or DC) with a new episode of back pain between April 2015 and March 2020. Back pain was defined as pain located in the region from the top of the scapulae to the first sacral vertebrae. A new episode was defined as being preceded by 6 months without visiting a primary care provider for a similar complaint. Patients were excluded from the study if they had a cognitive impairment which precluded them from completing the study questionnaires or if they had difficulties speaking and writing Norwegian. Patients who had severe mobility impairments (i.e., were wheelchair bound) were excluded as they would not be able to complete the physical examination.

Participants were recruited from primary care practices across Mid- and Southern Norway, including both cities and rural areas. The patients were invited to participate in the study by their health care practitioner. Those who fit the eligibility criteria and completed an informed consent to participate-form, responded to a comprehensive questionnaire and underwent a standardized physical examination at baseline by one of the study coordinators were included. We did not collect data on eligible individuals who were not included. The study coordinators were physiotherapists or chiropractors given standardized training in the examination procedure.

### Data collection

**Fig. 1:**
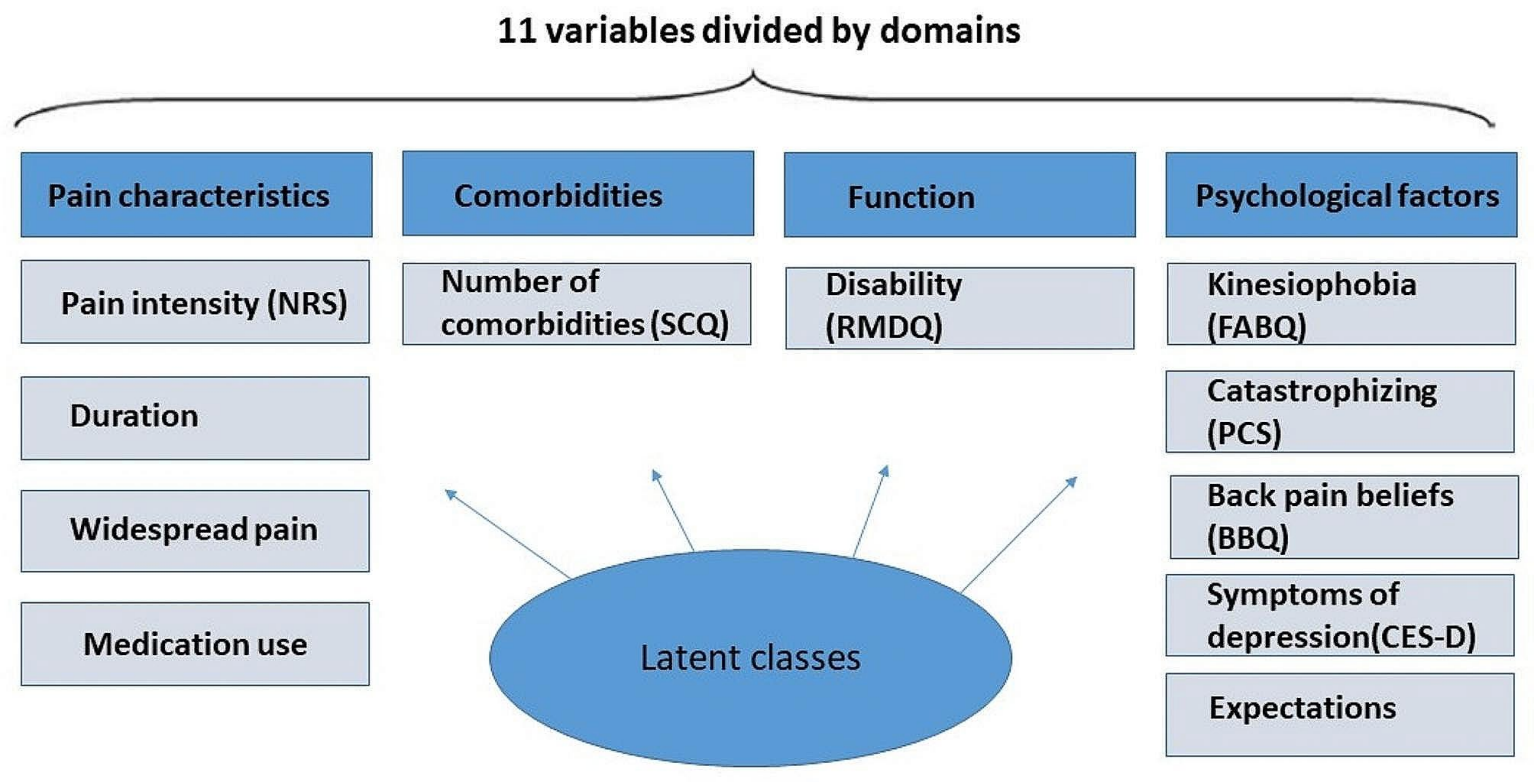
Overview of the variables in the latent class analysis. NRS, Numeric Rating Scale; SCQ, Self-administered Comorbidity Questionnaire; RMDQ, Roland-Morris Disability Questionnaire; FABQ-PA, Fear-Avoidance Beliefs Questionnaire-Physical Activity subscale; PCS, Pain Catastrophizing Scale; BBQ, Back Beliefs Questionnaire; CES-D, Centre for Epidemiological Studies-Depression

The questionnaire and history taking during the inclusion (baseline) visit included questions on patient characteristics, characteristics of the back complaint, medication consumption, function, psychological factors and comorbidities. A selection of these variables was used for this analysis (Fig. 1). The physical examination comprised general examination of the body, range of motion of the back and hips and additional orthopedic and neurological tests. Details of additional data can be found elsewhere. Follow-up questionnaires were sent at 3, 6, 12, and 24 months after inclusion, but paper versions were available for participants who were unfamiliar with electronic data collection. While the study was ongoing, patients received care as usual.

### Variables in the latent class modelling

Based on previous research, eleven indicator variables were extracted from the dataset and used in the analysis (Fig. 1). These include (1) pain characteristics: intensity (measured by the Numeric Rating Scale (NRS, range 0–10, higher scores indicate higher pain intensity), duration of current complaint (0–14 days, 15–90 days, 91–365 days or ≥ 366 days), widespread pain (measured by the pain drawing from McGill Pain Questionnaire and the revised criteria from Wolfe et al. for widespread pain [39]), medication consumption for back pain (yes or no); (2) function: disability (measured by Roland-Morris Disability Questionnaire (RMDQ, range 0–24, higher scores indicate more back-related disability); (3) comorbidities: number of comorbidities (measured by Self-administered Comorbidity Questionnaire, range 0–7); (4) psychological factors: kinesiophobia (measured by the physical activity subscale of the Fear-Avoidance Beliefs Questionnaire, range 0–24, higher score indicates higher levels of kinesiophobia), pain catastrophizing (measured by Pain Catastrophizing Scale, range 0–52, higher score indicates more pain catastrophizing), back beliefs (measured by Back Beliefs Questionnaire, range 9–45, where that a high score indicates more pessimistic beliefs regarding the consequences of back pain), symptoms of depression (measured by Centre for Epidemiologic Studies-Depression questionnaire, range 0–60, higher scores indicates the presence of more symptomatology) and expectations (expectations of their back pain in three months, better/much better or no change/worse).

### Statistical analysis

Descriptive statistics were performed in IBM SPSS Statistics Version 26 for Windows [40]. A single-stage LCA, modelling all variables simultaneously, was conducted in MPlus version 8.3 [41]. The model with the best fit was selected by comparing several fit indices and making a choice based on multiple aspects. For descriptive model comparisons the information criteria (BIC, AIC) were explored, where lower values indicate better model fit [42, 43]. Furthermore, mean posterior probability values were examined. These should be equal to or larger than 0.8, indicative of low levels of misclassification. The quality of the classification in the models was additionally inspected by the entropy value, where values close to 1 indicate good classification accuracy and little ‘fuzziness’ [44]. After the choice for the final model was made, patients were allocated to their best fitting class.

### Transformation of data

The variable “duration” was categorized into the following categories: 0–14 days, 15–90 days, 91–365 days, and “366 days and more”. No data were imputed as the likelihood approach used in LCA includes a procedure for handling missing values and does not require complete data [44, 45].

### Model selection

An explorative and common, forward approach to the model specification was performed [46], i.e., classes were added to the model until the model did not improve any further. The clinical interpretability between the classes was also inspected.

First, the models were compared using information criteria (IC)-based fit statistics. These include the Bayesian Information Criteria (BIC; [43]), Akaike Information Criteria (AIC; [42]), and Adjusted BIC [47]. Second, entropy statistics were used as a marker of the accuracy with which models classified individuals into their most likely class. Patients´ subgroup membership was assessed by the average posterior probabilities. Lastly, the preferred models were compared by inspecting the clinical interpretability and the respective number of individuals in each subgroup [48] and then labeled according to their distinct characteristics.

### Health care providers

To explore if individuals in the identified classes sought different health care providers as their first point-of-contact, a cross-table was constructed, and X^2^-tests performed to compare the observed and expected proportions of patients who were seeking care with GPs, PTs and DCs across classes. The results are presented as the estimated proportions of patients seeking a given type of first point-of-contact for each class. All the point estimates are presented with 95% confidence intervals (CI).

## Results

### Description of the Sample

In total, 435 patients were included in this study, 28% were recruited from GPs, 29% from PTs and 43% from DCs. The median age was 66 (IQR: 59–72) and around half of the patients were women (53.1%). The vast majority, 94.6%, had experienced back pain before this current episode. The majority of the sample reported pain in the lumbar/ lower spinal region only (85%), 4% had pain only in the thoracic level and 43% reported having radiating (uni- or bilateral) leg pain the previous week.

For the total sample, all scores of the psychological variables were below the cut-off values for clinical symptoms. For example, the median score of the CES-D for depressive symptomatology was 8.0 (IQR; 3–13), whereas the cut-off point of 16 or above are considered identifying those at risk of clinical depression [49]. Also, three out of four had high expectations of recovery, expecting to be fully recovered or much better within 3 months.

**Fig. 2:**
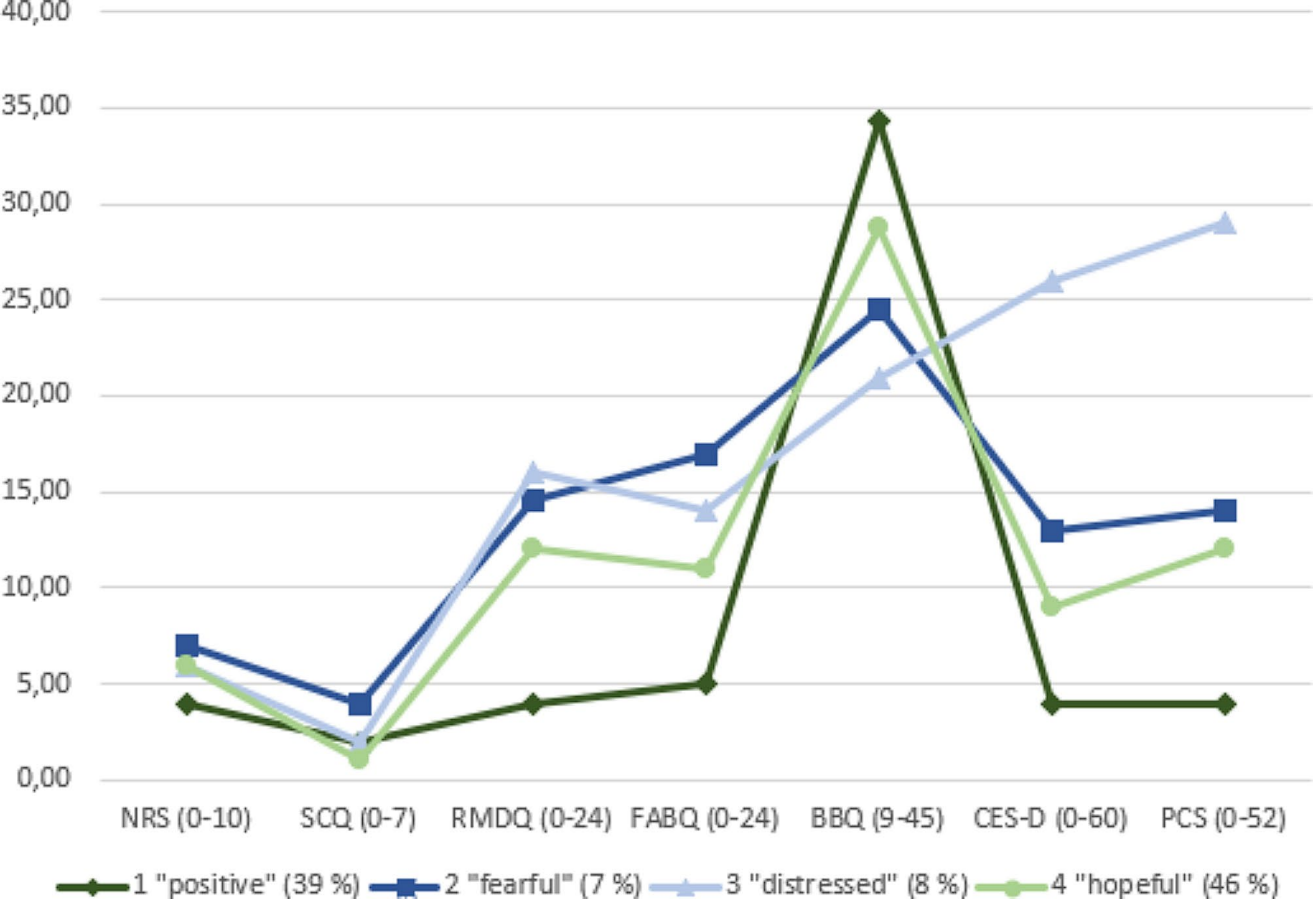
Mean scores of each class’ questionnaire values, class size in brackets. NRS, Numeric Rating Scale; SCQ, Self-administered Comorbidity Questionnaire; RMDQ, Roland Morris Disability Questionnaire; FABQ, Fear-Avoidance Beliefs Questionnaire; BBQ, Back Beliefs Questionnaire; CES-D, Centre for Epidemiologic Studies-Depression questionnaire; PCS, Pain Catastrophizing Scale

The latent class analysis identified a model with four classes as the optimal fit. The average posterior probabilities for classes 1 to 4 were 0.905, 0.858, 0.961, and 0.877 respectively. The characteristics of the classes are shown in Table 1 and Fig. 2.

In class 1 (*n* = 169, 39%), labeled the “positive” class, the mean age was 66 years and half were men, and the group had the highest proportion of employed individuals. The mean pain intensity level for this group was 3.92/10, and less than one out of five were taking pain medication for their back pain. This class also showed lower functional disability, kinesiophobia, pain catastrophizing, and symptoms of depression and high positive beliefs about back pain compared to the other classes.

For class 2 (*n* = 31, 7%), labeled the “fearful” class, the mean age was the highest among the groups, 72.3 years, and a high proportion (71%) were women. The individuals in this group had the most comorbidities of all classes. The mean pain intensity was 6.9/10, and nearly two out of three took pain medication for their back pain. The median disability score (14.50) of this group was the second highest of all groups. They also scored the second highest on symptoms of depression and pain catastrophizing.

Class 3 (*n* = 33, 8%), labeled the “distressed” class, had a mean age of 62.8 years and a high proportion (70%) were women. The mean pain intensity score was 6.1/10, and nearly three out of four took pain medication for their back pain. This class had the highest proportion of individuals with widespread pain (18.2%) and the highest score on functional disability of the classes. For symptoms of depression, the median score of this subgroup was 26.0 on the CES-D scale, well above the cut-off point for symptoms of clinical depression. Out of all four classes, this class also had the highest score of pain catastrophizing and the most negative back pain beliefs.

Class 4 (*n* = 202, 46%), labeled the “hopeful” class, was the largest class by number, making up nearly half of our sample (46,4%), and had a mean age of 66.6 years. Half of the group were women, and just under half of the individuals had higher education. This class had the lowest number of comorbidities. The mean pain intensity score was 6.1/10, and their pain related disability was 12/24 on the RMDQ. Over 80% of the individuals in this group believed their back pain would improve in the next three months.

**Table 1:** Characteristics of the full sample and stratified by class

	Total sample (*n* = 435)	The positive (*n* = 169), 39%	The fearful (*n* = 31), 7%	The distressed (*n* = 33), 8%	The hopeful (*n* = 202), 46%
**Sociodemographics**					
Age (y), mean (SD)	66.0 (13.0)	66.0 (7.7)	72.3 (9.6)	62.8 (8.4)	66.6 (8.3)
Women, n (%)	231 (53.1)	84 (49.7)	22 (71.0)	23 (69.7)	102 (50.5)
Educational level, n (%)					
Low	244 (56.5)	87 (51.5)	14 (45.2)	22 (68.8)	121 (60.5)
High	188 (43.5)	82 (48.5)	17 (54.8)	10 (31.3)	79 (39.5)
Employed, n (%)	201 (46.2)	87 (51.5)	7 (22.6)	10 (30.3)	97 (48.0)
Comorbidities, > 4, N (%)	34 (10.2)	6 (3.6)	24 (77.4)	3 (10.3)	1(0.6)
Intake of medications for back pain, n (%)	165 (39.9)	28 (17.6)	20 (64.5)	23 (74.2)	94 (48.7)
**Pain characteristics**					
Pain intensity, mean (SD)	5.3 (2.30)	3.9 (2.3)	6.9 (2.1)	6.1 (1.7)	6.1 (2.0)
Duration of current back pain episode, n (%)					
0–14 days	150 (39.9)	68 (47.2)	9 (37.5)	13 (44.8)	60 (33.5)
25-90 days	151 (40.2)	54 (37.5)	4 (16.7)	8 (27.6)	85 (47.5)
91-365	46 (12.2)	15 (10.4)	7 (29.2)	4 (13.8)	20 (11.2)
≥366 days	29 (7.7)	7 (4.9)	4 (16.7)	4 (13.8)	14 (7.8)
Widespread Pain, n (%)	30 (6.9)	7 (4.1)	4 (12.9)	6 (18.2)	13 (6.4)
**Disability**					
RMDQ score, median (IQR)	9.0 (4–13)	4.0 (2–6)	14.5 (12–17)	16.0 (12–19)	12.0 (8–14)
**Psychological factors and pain related behavior**					
FABQ score, median (IQR)	9.0 (5–13)	5.0 (0.5-9)	17.0 (14–20)	14.0 (10-17.5)	11.0 (7–14)
CES-D score, median (IQR)	8.0 (3–13)	4.0 (1-7.3)	13.0 (10–18)	26.0 (20–30)	9.0 (5–13)
**Back pain related beliefs and attitudes**					
PCS score, median (IQR)	9.0 (4–15)	4.0 (1-6.5)	14.0 (10–19)	29.0 (23-37.5)	12.0 (7–16)
BBQ score, mean (SD)	23.8 (7.1)	19.7 (5.4)	29.5 (6.8)	33.1 (7.2)	25.3(5.9)
Expectations, n (%)					
Fully recovered or much better	328 (75.7)	122 (72.2)	21 (70.0)	23 (69.7)	162 (80.6)
No change or worse	105 (24.3)	47 (27.8)	9 (30.0)	10 (30.3)	39 (19.4)

RMDQ, Roland Morris Disability Questionnaire; FABQ, Fear-Avoidance Beliefs Questionnaire; CES-D, Centre for Epidemiologic Studies-Depression questionnaire; PCS, Pain Catastrophizing Scale; BBQ, Back Beliefs Questionnaire

In Table 2, the first point-of-contact for individuals in each class is reported. The proportions of patients suggest that the fearful and distressed were mainly consulting a GP, the classes were evenly distributed for patients consulting a PT and patients in the positive and hopeful classes were mainly consulting a DC. The only statistically significant association between type of first point-of-contact and a given class was found for the “positive” class where the proportion of patients visiting a chiropractor was significantly higher compared to the proportions of patients seeking GPs and PTs.

**Table 2:** First point-of-contact for the sample and each class, reported as numbers and proportions to investigate if the identified classes differed in terms of type of health care provider

Professional	Full sample, N (%)	The positive, N	The estimated proportion, % (95% CI)	The fearful, N	The estimated proportion, % (95% CI)	The distressed, N	The estimated proportion, % (95% CI)	The hopeful, N	The estimated proportion, % (95% CI)
GP	123 (28.4)	32	19.2 (13.5 to 26.1)	14	45.2 (27.3 to 64.1)	14	42.4 (25.5 to 60.8)	63	31.2 (24.9 to 38.1)
PT	126 (29.1)	45	26.9 (20.4 to 34.3)	9	29.0 (14.2 to 48.0)	11	33.3 (18.1 to 51.8)	61	30.2 (24.1 to 37.0)
DC	184 (42.5)	90*	53.9 (46.0 to 61.6)	8	25.8 (11.9 to 44.6)	8	24.2 (11.1 to 42.3)	78	38.6 (31.9 to 45.7)

The confidence level for the class distribution is reported as lower and upper bounds

GP, General Practitioner; PT, Physiotherapist; DC, Chiropractor; CI, confidence interval

*Statistically significant at the 0.05 level

## Discussion

This study identified four distinct classes among individuals aged 55 or older seeking primary care for their back pain, based on 11 key prognostic factors, including pain characteristics, comorbidities, disability and psychological factors. The classes were distinctly different in terms of severity of symptoms (pain intensity, functional disability, use of medication), comorbidities, and psychological characteristics, and a higher proportion of “positive” patients visited a chiropractor as first point-of-contact.

The majority of individuals (classes 1 and 4, 371 individuals, 85%) may be labelled as having favorable psychological and behavioral characteristics, according to our selected variables. Thus the sample seemed to be in good shape in terms of pain, psychological and behavioral characteristics.

The largest class, number 4, the “hopeful” patients, were characterized by high expectations to pain improvements in the upcoming months. Despite relatively high pain intensity (6.1/10), these patients had high expectations for improvement. This was echoed in the second-largest class, number 1, the “positive” class, consisting of individuals who reported low scores on all the variables, except expectations for a positive outcome, thus they did not have any predictors for a negative outcome.

Classes 2 and 3 were considerably smaller, with 31 and 33 individuals, respectively. Despite the group sizes, they were supported by the statistical model selection values/criteria (among others AIC, BIC, posterior probabilities and entropy). Individuals classified in classes 2 and 3 generally had poor scores on all the variables measured, thus were clearly “unhealthier” than the majority of individuals in classes 1 and 4. Class 3 stands out from group 2 by its higher scores on symptoms of depression and pain catastrophizing, which are well known prognostic factors for persistent back pain [32].

Most of the individuals in this study were recruited from chiropractors’ offices, but this does not necessarily reflect the pattern of first point-of-contact for back pain among the elders. Overall, GPs, PTs and DCs see the same proportion of positive, distressed, fearful and hopeful older adult patients with back pain, with the only difference being a higher proportion of the “positive” patients sought a chiropractor as their first point-of-contact compared to the proportions of patients who sought a GP or PT.

To the best of our knowledge, no previous studies have investigated classes among the older adult seeking care for back pain. A Norwegian study on people of working ages (up to 67 years) on patients seeking care for musculoskeletal pain identified five latent classes, also based on pain and psychological factors [29]. These five classes showed, in line with our study, a “graded” approach, from the very severely affected to the lightly affected patients. However, our sample (apart from being older) suffered from pain of shorter duration and of higher intensity, possibly reflecting the inclusion criteria of not having sought care the previous six months, thus not including individuals with persistent pain.

In a Danish study, adults (up to 65 years) seeking chiropractic care for low back pain were included if not having needed more than one previous appointment for their pain in the previous three months [24]. This study used a single-stage and a two-stage approach and identified seven and nine classes, respectively, based on a wide range of pain, psychological, functional, participatory and impairment factors. The resulting classes were described in terms of pain characteristics (duration, intensity and radiation) as well as impact on work and sleep and are therefore not easily comparable.

In a German study, patients receiving multimodal treatment for chronic pain, aged between 18 and 86 years, were included [50]. Four classes, based on pain characteristics and health data, were identified. As in our study, these ranged from a group with “high pain burden” to a group with “low pain burden”, with the severely affected representing the majority of the sample. Thus, it is likely that these classes are common across ages, but that the proportion severe/lightly affected differs between populations. However, for the older population, we need to explore if these challenges translate into a poorer outcome. If so, interventions could be directed towards specific classes.

In a previous publication with data from the same cohort, differences between individuals seeking care with a GP, PT or DC were explored [17]. It was found that patients with more severe pain (longer duration and higher intensity) were likely to visit the GP or PT, whilst those with high expectations of recovery and widespread pain were likely to visit the DC. We found that among the “positive” a higher proportion sought chiropractic care compared to the proportions of patients seeking a GP or PT. However, for the people in the “hopeful” class the probabilities of seeking the three types of first contact point were similar. In the latent classes, the highest proportion of widespread pain was found in the “hopeful” class. These differences are likely due to the fact that our classes were based on many variables. Thus, the identified classes encompass a broader picture of the pain experience as well as patient characteristics.

This study has some strengths. We recruited patients from multiple primary care clinics in both urban and rural parts of Norway, strengthening the external validity of our results. We also had a relatively large sample size and good quality data, few missing datapoints, as we used validated questionnaires. The method, LCA, is data-driven, but based on previously identified prognostic variables for back pain outcome. We a priori included variables that cover the most expected factors for treatment outcomes in this patient group [32–36]. To decide the number of groups, we utilized statistical criteria alongside clinical knowledge, further strengthening the relevance of the identified classes.

Some limitations should be considered. The classes were based on variables that were selected according to existing prognostic research. It cannot be ruled out that the classes may have looked different if we had included other variables or had used prospective data instead of cross-sectional. However, the similarity of the identified classes with those of other studies of younger individuals, which have included slightly different selections of variables, suggests that this is not the case.

We did not have information regarding excluded patients mainly to minimize the burden on the recruiting clinicians, as previously described [17]. Likely, this has resulted in selection bias, as many individuals with persistent pain were excluded due to the criterion of not having sought care the past six months, leaving us with a cohort with favorable psychological and behavioral characteristics, not necessarily representative of the older adult population at large. We have previously compared the BACE-N sample to a representative general population sample with musculoskeletal disorders and found that the BACE-N sample is likely over-represented by men, those with higher education, and those in paid work [17], which may explain why the majority of our sample had favorable psychological and behavioral characteristics. Further, we have not performed an external validation of our classes, thus we don’t know if the classes would have been generalizable. However, a Norwegian study on common musculoskeletal disorders somewhat similar to our study found low classification error and comparable classes when performing an external validation [29].

## Conclusions

This study identified four classes among individuals aged 55 or older seeking primary care for their back pain; named the “positive”, “fearful”, “distressed” and “hopeful”. The classes were distinctly different in terms of severity of symptoms (pain intensity, functional disability, and use of medication), comorbidities, and psychological characteristics. The patients in the “positive” class were more likely to use chiropractors as their first point-of-contact compared to the other classes.

Even though the identified classes appear to have clinical relevance, it remains to be explored if the individuals also develop differently over time.

### Electronic supplementary material

Below is the link to the electronic supplementary material.

**Supplementary table 1**: Fit-indices, entropy and PPs for all the tested models.

## Data Availability

All data relevant to the study are included in the article or uploaded as online supplemental information.

## Abbreviations

MSK: musculoskeletal
BACE: Back complaints in elders
LBP: low back pain
LCA: Latent Class Analysis
GP: general practitioner
PT: physiotherapist
DC: doctor of chiropractic
NRS: numeric rating scale
RMDQ: Roland-Morris Disability Questionnaire
FABQ: Fear-Avoidance Beliefs Questionnaire
PCS: Pain Catastrophizing Scale
BBQ: Back Beliefs Questionnaire
CES: D-Centre for Epidemiologic Studies-Depression questionnaire
