# Supplementary material for: Identifying latent subgroups in the older population seeking primary health care for a new episode of back pain – findings from the BACE-N cohort
Source: BMC Musculoskelet Disord. 2024 Jan 13;25:60. doi: 10.1186/s12891-024-07163-0 (PMC10787445; doi:10.1186/s12891-024-07163-0)
Supplement: Supplementary file 1 — Supplementary table 1: Fit-indices, entropy and PPs for all the tested models. [file 12891_2024_7163_MOESM1_ESM.docx]

Supplementary table 1: fit-indices, entropy and PPs for all the tested models.

|  | 1-class model | 2-class model | 3-class model | 4-class model | 5-class model |
| --- | --- | --- | --- | --- | --- |
| AIC | 19650.06 | 19125.70 | 18965.04 | 18897.93 | 18847.18 |
| BIC | 19739.72 | 19280.56 | 19185.11 | 19183.21 | 19197.66 |
| Sample-size adjusted BIC | 19669.94 | 19159.97 | 19013.74 | 18961.06 | 18924.74 |
| Entropy |  | 0.83 | 0.77 | 0.81 | 0.76 |
| PP | 0.905 | 0.858 | 0.961 | 0.877 |  |

AIC: Akaike information criterion

BIC: Bayesian Information Criteria

PP: Posterior probabilities
